# The Role of Hepatocyte Growth Factor (HGF) in Insulin Resistance and Diabetes

**DOI:** 10.3389/fendo.2018.00503

**Published:** 2018-08-30

**Authors:** Alexandre G. Oliveira, Tiago G. Araújo, Bruno de Melo Carvalho, Guilherme Z. Rocha, Andrey Santos, Mario J. A. Saad

**Affiliations:** ^1^Department of Internal Medicine, State University of Campinas, Campinas, Brazil; ^2^Department of Physical Education, Institute of Biosciences, São Paulo State University (UNESP), Rio Claro, Brazil; ^3^Department of Physiology and Pharmacology, Federal University of Pernambuco, Recife, Brazil; ^4^Institute of Biological Sciences, University of Pernambuco, Recife, Brazil

**Keywords:** hepatocyte growth factor, HGF, obesity, insulin resistance, diabetes, inflammation, beta cells

## Abstract

In obesity, insulin resistance (IR) and diabetes, there are proteins and hormones that may lead to the discovery of promising biomarkers and treatments for these metabolic disorders. For example, these molecules may impair the insulin signaling pathway or provide protection against IR. Thus, identifying proteins that are upregulated in IR states is relevant to the diagnosis and treatment of the associated disorders. It is becoming clear that hepatocyte growth factor (HGF) is an important component of the pathophysiology of IR, with increased levels in most common IR conditions, including obesity. HGF has a role in the metabolic flux of glucose in different insulin sensitive cell types; plays a key role in β-cell homeostasis; and is capable of modulating the inflammatory response. In this review, we discuss how, and to what extent HGF contributes to IR and diabetes pathophysiology, as well as its role in cancer which is more prevalent in obesity and diabetes. Based on the current literature and knowledge, it is clear that HGF plays a central role in these metabolic disorders. Thus, HGF levels could be employed as a biomarker for disease status/progression, and HGF/c-Met signaling pathway modulators could effectively regulate IR and treat diabetes.

## Introduction

Obesity is a rapidly growing worldwide epidemic. It is estimated that more than 1 billion adults worldwide are obese (BMI ≥ 30 Kg/m^2^) or overweight (BMI between 25 and 30 kg/m^2^) (1–3). It is projected that by 2025, 40% of the population in the US, 30% in England and 20% in Brazil will be affected by obesity (1–3). The WHO has declared that obesity is a key factor in the development of type 2 diabetes (1). Along these lines, there are a plethora of studies that have investigated the roles of and relationships among nutrition, physical activity and genetic susceptibility as determinants of the current prevalence of obesity. Additionally, it is well established that there is a direct relationship between obesity, the onset of insulin resistance (IR) and diabetes.

Insulin resistance is manifested by reducing the ability of insulin to activate the insulin signaling pathway (4, 5). At the molecular level, IR is characterized by diverse alterations in various intracellular signaling pathways. In fact, it has been shown that insulin signaling is impaired in the liver, muscle, adipose tissue, hypothalamus, and others tissues, in IR states (6, 7). While there are several biological events that can lead to the impairment of the insulin signaling pathway, chronic inflammation is perhaps the best described. Inflammation promotes serine phosphorylation of insulin receptor substrate (IRS), through the action of serine kinases such as c-Jun N-terminal kinase (JNK) and inhibitor of kappa B kinase (IKKβ) (6, 8, 9).

Diabetes is another major global health problem, from which the social and economic consequences are devastating. For example, this disease is directly or indirectly responsible for approximately 4 million deaths per year, which corresponds to 9% of the total world mortality (10). In 2013, the International Diabetes Federation (IDF) reported that diabetes affected 382 million people worldwide, and this number is expected to grow to 592 million, by 2035 (10, 11).

Obesity, IR and type 2 diabetes are closely related to sub-clinical and chronic inflammation, which is characterized by abnormal cytokine production, including an increase in acute phase molecules and other mediators, as well as inflammatory signaling pathway activation (6, 12, 13).

Within this inflammatory context, the liver may have an important contribution, because during overeating, which is common in obesity, it expresses and secretes several inflammatory proteins (14, 15). The liver may also play an important role in IR (16). Likewise, in obesity-induced IR, the suppression of hepatic glucose production becomes impaired, contributing to the development of hyperglycemia. Furthermore, the liver participates in metabolic regulation, through the expression and secretion of organokines, which are also referred to as hepatokines. Interestingly, these proteins influence metabolic processes through paracrine and endocrine signaling, rather than by autocrine signaling (17). Hepatokines include several growth factors such as fibroblast growth factor 21 (18), insulin-like growth factors, angiopoietin-related growth factor, and HGF. The characteristics and roles of the latter will be the focus of this review.

HGF synthesis and secretion are up-regulated in IR states, and is thought to be an important component of the pathophysiology of IR (19–30). Interestingly, the receptor of HGF, c-Met, is structurally related to the insulin receptor (INSR) (31). Furthermore, HGF signaling participates in the modulation of the metabolic flux of glucose in different insulin sensitive cell types such as β-cells, enterocytes, adipocytes, hepatocytes and myocytes (25, 31–36). HGF signaling also plays a key role in β-cell homeostasis (33, 37–47). Lastly, HGF has been shown to participate in the modulation of the inflammatory response (48–51). This review discusses the significance of each of these five HGF features in the pathophysiology of IR and diabetes and presents evidence for HGF in the etiology of cancer.

## Structure and molecular biology of HGF and its receptor (c-Met)

**Figure 1:**
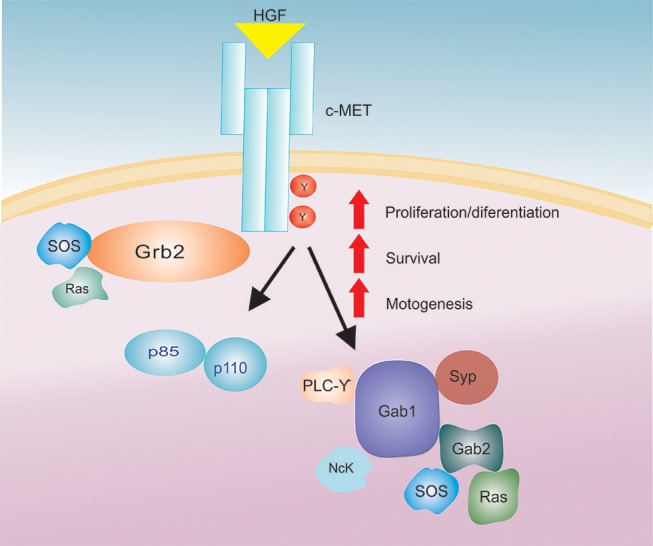
The HGF/c-Met signaling axis. After HGF binding to the c-Met receptor there is an activation of a signaling cascade that increases several biological actions (proliferation/differentiation, survival and motogenesis).

Originally, HGF was identified as a liver-regenerative circulating factor, since levels of this protein increased following liver injury or hepatectomy (47, 52, 53). Currently, HGF is recognized as a molecule with diverse activities, and has been demonstrated to have mitogenic, morphogenic, and motogenic effects in different tissues (Figure 1) (54).

The HGF locus is harbored on chromosome 7q21.1, covering approximately 70 kb and consists of 18 exons and 17 introns (55). The translation of a single 6 kb transcript results in a pre-pro-polypeptide of 728 amino acids (56). In the liver, HGF is synthesized and secreted as an inactive single chain precursor (proHGF, 92 kDa). The active form of HGF is produced by proteolytic cleavage of proHGF, between Arg^494^ and Val^495^. Active HGF consists of a heavy chain (62 kDa) and a light chain (32–36 kDa), which are covalently linked by a disulfide bridge (57, 58). *In vitro* studies have shown that blood coagulation factor XIIa, urokinase, plasminogen activator and HGF activator can all activate HGF. Among these proteases, HGF activator (34 kDa) is the most efficient at converting proHGF into mature HGF (59). Both the liver and, to a lesser extent, gastrointestinal tissue synthesize HGF activator (60, 61). Following translation, this protease circulates as an inactive precursor, activator proHGF (96 kDa), and is activated upon thrombin cleavage (62). These studies highlight the important role HGF activator plays in regulating HGF activity. Moreover, a HGF activator inhibitor (HAI-1) was recently isolated, and acts as a physiological HGF activator inhibitor at the cell surface (53, 63). However, its role needs to be better elucidated.

Biological activities (i.e., proliferation/survival/motogenesis) mediated by HGF binding to the c-Met receptor require the active form of HGF. This receptor is encoded for by the c-met protooncogene (p190^MET^) (33). Additionally, the c-Met receptor is a dimer composed of an alpha-chain (50 kDa), and a beta-chain (145 kDa), which are linked together by a disulfide bond. The alpha chain is highly glycosylated and exposed on the cell surface, while the beta-chain is a transmembrane protein containing the kinase domain, the tyrosine autophosphorylation site and the multifunctional binding site (53, 64). The c-Met receptor is more closely related to the INSR, in terms of the overall structure of the protein and the sequence of the kinase domain, than any other member of the receptor tyrosine kinase (RTK) subclasses (65).

Upon HGF binding, c-Met activation and subsequent signal transduction rely on the presence of adenosine triphosphate (ATP) and Mg^2+^ ions, which promote receptor dimerization and trans-phosphorylation of the catalytic domain. Additionally, HGF binding to c-Met and subsequent phosphorylation of intracellular transducers such as: PI3K (p85 subunit), Gap, Ras, PLC-gamma, Src related tyrosine kinase, Grb-2, Gab-1, IRS-1, IRS-2 (Figure 1) (33, 53, 56, 66) has been shown to promote HGF functions in the cell.

**Figure 2:**
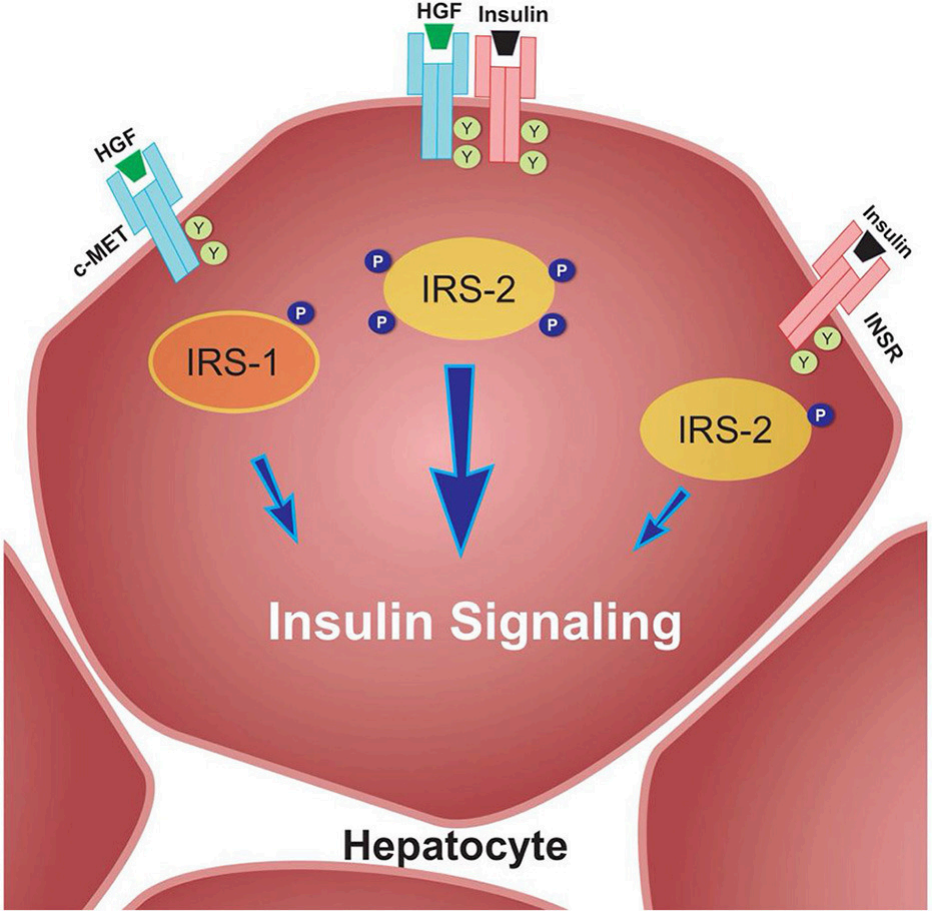
c-Met/INSR crosstalk in hepatocytes. The c-Met/INSR complex increases the recruitment of IRS-1 and 2 and thereby amplifies the insulin signaling pathway in hepatocytes. Adapted from Fafalios et al. (31).

For example, Fafalios et al. (31) demonstrated that c-Met activation in the liver induces the formation of a c-Met-INSR complex, which stimulates the recruitment of insulin receptor substrates (IRS-1 and IRS-2) and thereby amplifies insulin signaling (Figure 2). Furthermore, this study showed that activation of this complex improves hepatic glucose metabolism, by increasing glucose uptake and decreasing hepatic glucose production (31). These findings reinforce the notion that HGF-induced signaling, at least in the liver, imparts protection against IR. These effects need to be further elucidated in both the liver and other metabolic tissues in an IR state.

## The role of HGF in the modulation of glucose flux in different insulin sensitive cell types

Several studies have demonstrated that HGF has regulatory effects on glucose transport and metabolism. One such study showed that HGF overexpression, in murine β-cells, increases the transcription of GLUT-2 and glucokinase, leading to increased glucose uptake and metabolism in these cells (32). On the other hand, the ablation of HGF/c-Met signaling, in adult murine β-cells, led to a decrease in GLUT-2 expression, followed by glucose intolerance and an impairment in glucose-stimulated insulin secretion (33). Additionally, HGF was shown to interfere with the function of the Na^+^/glucose cotransporter and GLUT-5, in rat intestinal cells (34). Furthermore, HGF promotes the translocation of GLUT4 to the cell membrane and increases glucose uptake through the activation of PI3K, in 3T3-L1 adipocytes (25). With regards to regulating glucose transport and metabolism in the liver, it has been demonstrated that HGF is able to stimulate hepatic glucose uptake and suppress hepatic glucose output (31). In fact, an *in vitro* study demonstrated that HGF substantially increases glucose transport and metabolism in myotubes, through a mechanism mediated by the PI3K/Akt pathway, resulting in increased GLUT-1 and GLUT-4 plasma membrane expression (35). More recently, the same group presented further evidence of HGF participation in skeletal muscle glucose homeostasis (36). In this study, a transgenic model, capable of increasing skeletal muscle HGF levels by three-fold, without altering plasma HGF levels was employed. When these transgenic mice were fed a high-fat diet there was an observed improvement in glucose tolerance, and an increase in Akt phosphorylation levels in the gastrocnemius muscles (36). Thus, reinforcing the notion that HGF plays an important role in obesity-mediated IR in muscle.

## Regulation of pancreatic islet β-cell homeostasis by HGF

There is a great interest in identifying endogenous regulatory factors that control both β-cell mass expansion and insulin secretion. In β-cell cultures, Otonkoski et al. (40) investigated the mitogenic and morphogenic activity of various growth factors, and were the first to observe the insulinotropic activity of HGF. Later, Hayek et al. (42) showed that adding HGF to culture medium induced β-cell proliferation. Furthermore, it was observed that mRNAs, encoding for HGF and c-Met receptor, are highly expressed in pancreatic β-cells during early development, and remain at low levels during puberty and throughout adult life (41). These studies have raised interest in identifying HGF as a potential element in the development of hyperplasia in β-cells, and subsequent hyperinsulinemia.

**Figure 3:**
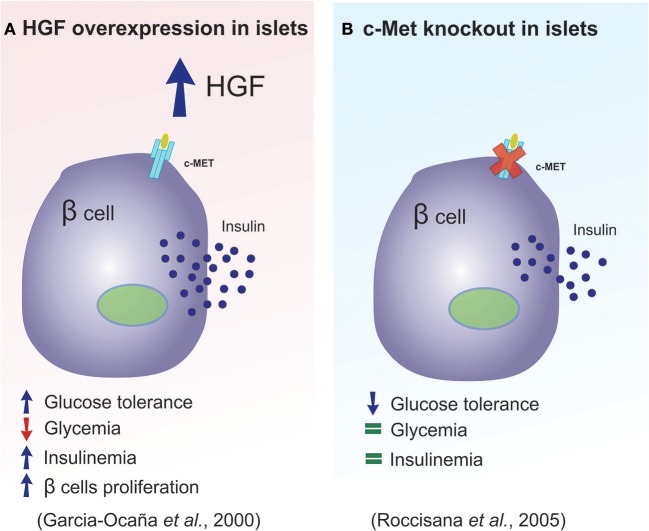
HGF plays a key role in pancreatic β-cell homeostasis. **(A,B)** Diagram illustrating the main differences observed between the overexpression of HGF and the absence of the c-Met receptor in pancreatic β-cells.

In 2000, Garcia-Ocana et al. developed transgenic mice that overexpressed HGF, with the intention of studying the role of this protein on the pancreatic islets, *in vivo*. The authors concluded that *in vivo* HGF overexpression increased β-cell proliferation, the number of islets, β-cell mass, as well as insulin production (47). Together these effects resulted in animals that displayed moderate hypoglycemia and hyperinsulinemia (Figure 3A summarizes main results of this study). In 2005, two studies used a c-Met receptor knockout mouse model to investigate the physiological consequence of HGF inhibition in pancreatic β-cells, and the results were discordant in some aspects (33, 43). For example, Roccisana et al. (33) showed that c-Met receptor knockout animals presented a reduction in insulin secretion and a decrease in glucose tolerance, which was accompanied by a reduction in GLUT-2 expression in the β-cells. However, there were no observed changes in total β-cell mass, proliferation or islet morphology (33). The main findings of this research is illustrated in Figure 3B. On the other hand, Dai et al. (43) demonstrated that the size of the islets in this animal model was decreased, which was accompanied by reduced insulin content in the pancreas, lower insulin levels in the serum and mild hyperglycemia.

Furthermore, another study in mice demonstrated that the absence of c-Met, in the pancreas, led to a substantial increase in pancreatic β-cell death and a reduction in cell mass, which resulted in the manifestation of hypoinsulinemia. Consequently, these mice had a tendency to develop hyperglycemia in response to diabetogenic stimuli (46). Thus, these data indicate that HGF/c-Met-induced β-cell survival could be employed as a potential therapy for diabetes.

Another study from the Garcia-Ocaña group, demonstrated that HGF signaling is required for β-cell regeneration, following β-cell ablation (45). In this study, mice that underwent partial pancreatectomy (Ppx) and received a daily dose of HGF showed an increase in the rate of β-cell proliferation, when compared to animals that did not receive exogenous HGF. Conversely, c-Met knockout mice subjected to Ppx, had reduced β-cell mass, glucose intolerance, and decreased insulin secretion, when compared to wild type post-Ppx mice (45). More recently, a different mouse model was used to study the HGF-mediated regenerative effect (44). IRS2 KO mice are known to have an increased incidence of β-cell failure, increased apoptosis and reduced proliferation, which contributes to the development of diabetes in these mice (44, 67, 68). In order to investigate the effect of HGF on the observed β-cell failure, the IRS2 KO mice were cross-bred with transgenic mice overexpressing HGF in the β-cells. HGF overexpression was able to compensate for the negative effects related to the absence of IRS2, by normalizing β-cell mass and improving glucose homeostasis in the context of IR (44). Taken together, these findings indicate that HGF could be employed as a regenerative factor in the treatment of diabetes.

As mentioned above, the expression of c-Met in the β-cells results in enhanced HGF sensitivity, which can influence cell growth, survival and insulin secretion. Furthermore, HGF has both paracrine and endocrine properties, which are constantly regulating pathways involved in β-cell homeostasis. Following this line of reasoning, it was shown in hyperglycemia/diabetes, induced by stressful situations, that HGF has a protective role in physiological status of β-cells (37–39). Hence, HGF appears to be involved in the compensatory response of β-cells in two common conditions characterized by IR and associated with the pathophysiology of diabetes: (1) obesity, (2) pregnancy (37, 39). The latter has been shown to be directly associated with the development of gestational diabetes mellitus (GDM) (38).

In obesity, the most common insulin resistant condition, there is a compensatory pancreatic β-cell response against this hormonal resistance, which increases insulin secretion and maintains homeostatic glucose metabolism for a certain period of time. This compensatory β-cell response induces hyperinsulinemia, and is the first step in the development of diabetes (39). Since HGF stimulates insulin secretion and increases β-cell mass, both *in vitro* and *in vivo*, and due to the fact that increased circulating levels of this hormone are associated with obesity, HGF might link insulin resistance and β-cell hyperplasia. In fact, our group demonstrated through a dose-dependent, longitudinal approach that: (a) circulating HGF levels strongly correlate with β-cell mass increase in the IR state; (b) the β-cell mass increases in a HGF dose-dependent manner; (c) pharmacological blockage of the HGF signaling pathway abolishes the β-cell mass compensatory response; and (d) circulating HGF levels increase before the onset of the IR-induced β-cell compensatory response, thus suggesting a causal role in this specific event. Moreover, c-Met inhibition negatively affects the already impaired insulin signaling in the liver of diet-induced obese rats (37, 39). These HGF-mediated effects on β-cell proliferation and expansion indicate that this hormone has a crucial role in increased metabolic demand, which is commonly observed in obesity-induced IR.

Another adaptive β-cell mechanism, in which HGF participates, occurs during pregnancy. In this situation, the β-cell adapts to an increased demand for insulin and undergoes structural and functional changes, such as β-cell expansion and enhanced insulin secretion. However, any perturbations or disturbances in this adaptive process can lead to the development of GDM (69). Interestingly, while searching for potential therapeutic targets, for the treatment of GDM, it was found that HGF expression is upregulated in rat islet endothelium at gestational day 15 (70), which also happens to be when maximal β-cell proliferation is detected (38). Based on this evidence and the other roles of HGF in β-cell homeostasis, it was hypothesized that maternal β-cell adaptation is mediated by HGF. In fact, it was recently demonstrated that in the absence of c-Met, during pregnancy, there was a substantial impairment in β-cell proliferation and survival, resulting in β-cell mass reduction. In pregnant mice, this sequence of events culminates with hypoinsulinemia, consequent hyperglycemia and glucose intolerance, all of which are features of GDM (38). These observations implicate the HGF/c-Met signaling pathway in the pathogenesis of GDM, since ablation and inhibition of HGF or c-Met results in the disease. In this regard, targeting the HGF/cMet signaling pathway might represent a potential strategy for the treatment of GDM.

## Role of adipose tissue derived HGF

Obesity is associated with numerous metabolic changes, such as hyperinsulinemia and IR (71). The IR that occurs in adipose tissue may contribute to systemic IR and a variety of hepatic alterations (72, 73). Furthermore, secretion of adipokines, hormones, cytokines, chemokines, growth factors, acute phase proteins and HGF from the white adipose tissue (WAT) further complicates this condition (74). It is important to mention that even though HGF is considered a hepatokine, the synthesis and secretion of HGF has been demonstrated in murine 3T3-L1 adipocytes (20) and human adipose tissue (21, 22). Thus, it is quite plausible that the HGF synthesized in human adipose tissue plays an important role in obesity (23). Furthermore, due the importance of adipose tissue in IR, adipose-derived HGF expression might also have an influence on the obesity associated IR etiology. In fact, a recent study examined HGF secretion in different types of adipose tissue and observed that the HGF secretion pattern of the perivascular adipose tissue differs from the subcutaneous and visceral adipocytes (26). Surprisingly, this research also showed that, in both *in vitro* and *in vivo* models, perivascular fat cells have a much higher capacity for secreting adipokines, especially HGF, than other fat cell types. Additionally, it was shown that subjects with greater perivascular fat mass secreted more HGF (26), bringing to light the pathophysiological relevance of this hormone.

In humans, it has been demonstrated that levels of circulating HGF are elevated in obesity (27), metabolic syndrome (23), and diabetes mellitus (75). In obese individuals, circulating HGF levels were increased by more than 3-fold, when compared to lean individuals (27). HGF levels have also been correlated with anthropometric measures of obesity such as waist circumference, body mass index and body fat mass (24, 30). Furthermore, an association between weight loss induced by bariatric surgery and reduced HGF levels was previously observed, in obese patients (22, 28).

In patients with IR, a significant association between HGF and plasma insulin was observed suggesting a pathological link between them (23). A more thorough, 10-year prospective study proposed that elevated circulating levels of HGF were significantly associated with the development of IR (19). Furthermore, another prospective study, with over 12 years of follow-up, which included multi-ethnical individuals, also demonstrated that higher levels of serum HGF were positively associated with IR (76). Based on these data and the similarities between HGF and insulin, it is tempting to speculate that “HGF resistance” exists, which may contribute to glucose metabolism dysregulation. Finally, another study demonstrated that there is a significant correlation between circulating HGF levels and the Visceral Adiposity Index (VAI), which is a gender-specific mathematical index based on simple anthropometric and metabolic parameters as a presumed surrogate marker of adipose tissue function and distribution (29).

In summary, adipose tissue may be an important source of HGF, which may comprise an important component in adipose tissue dynamics. Thus, further emphasizing the role of HGF in the pathophysiological processes of obesity/IR/diabetes.

## The involvement of HGF in the modulation of the inflammatory response

Over the past 10 years, evidence has indicated that there is a correlation between IR and inflammation (6, 8, 77–79). In fact, subclinical chronic inflammation has been shown to be a major mechanism for the development of IR in peripheral tissues. This mechanism results in a chronic upregulation of pro-inflammatory cytokines (i.e., TNF-α and IL-6), which are known to exert deleterious effects on the insulin signaling pathway in adipocytes, hepatocytes, and myocytes (6, 8, 80). Furthermore, the actions of macrophages have been studied extensively, since they have been shown to accumulate in the liver and WAT, and promote IR and liver disease (81–83).

Studies have shown that HGF is involved in the modulation of the inflammatory response. Notably, Coudriet et al. demonstrated that HGF could decrease the acute phase of the inflammatory response by attenuating the production of IL-6, and increasing IL-10 levels, in lipopolysaccharide (LPS) stimulated bone marrow derived macrophages (48). Similarly, Flaquer et al., using *db/db* mice to study diabetic nephropathy, demonstrated that when the HGF gene was delivered to these animals, there was a reduction in the circulating levels of IL-6 and MCP-1, an increase in the number of M2 macrophages and an improvement in glomeruli inflammation (49).

**Figure 4:**
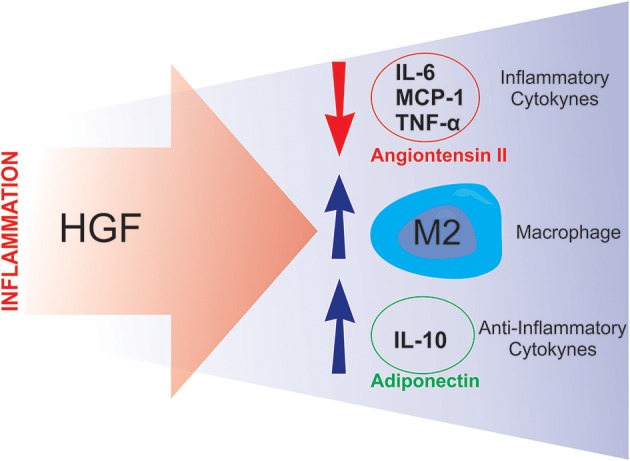
Putative compensatory role of HGF. During the inflammatory process, HGF seems to attenuate inflammation by inducing anti-inflammatory responses such as: downregulating angiotensin II activity and inflammatory cytokine synthesis, and stimulating M2 macrophage polarization and anti-inflammatory cytokine production.

Two studies from the Morishita group (50, 51), demonstrated novel HGF-induced anti-inflammatory mechanisms. The first study utilized a transgenic mouse-model that overexpressed HGF (HGF-Tg), which inhibited LPS-induced oxidative stress and vascular tissue inflammation. These HGF-mediated protective effects against the LPS-induced reactive oxygen species (ROS) production and inflammation were achieved via epidermal growth factor receptor (EGFR) degradation and angiotensin II (Ang II) signaling inhibition (50). The second study was divided into two stages: an *in vitro* study and an *in vivo* study. The *in vitro* stage of the study demonstrated that HGF disrupts NF-κB signaling in RAW264 macrophages, as well as in co-culture with 3T3-L1 adipocytes, leading to an overall reduction in the expression of MCP-1, TNF-α, and IL-6 (51). The second stage employed ApoE KO mice, which exhibit a chronic inflammation phenotype, including adipose tissue macrophage infiltration, adipocyte hypertrophy and fatty liver. After crossing the ApoE KO mice with HGF-Tg, the chronic inflammation, presented by the ApoE KO mice, was significantly reduced. The authors also detected an increase in serum adiponectin levels in the ApoE KO/HGF-Tg mice (51). Taken together, these data suggest that HGF suppresses the production of pro-inflammatory cytokines and, conversely, increases the secretion of adiponectin, thus breaking the macrophage-adipocyte inflammatory cycle (Figure 4). Furthermore, it has been recently shown that administering an antibody against HGF to wild type mice fed a high-fat diet, resulted in a more pronounced IR (84). A similar result was previously observed with pharmacological blockage of HGF signaling (37). Together, these results reinforce the already presented protective role of HGF on glucose metabolism in obesity. The observed results from HGF-Tg mice fed a high fat diet (HFD) also highlight the different anti-inflammatory effects of HGF in adipose tissue including the reduction in the levels of chemoattractants (MCP-1 and CXCL2), inflammatory cytokines (TNF-α and iNOS), as well as, pan macrophage marker (F4/80) (84). Thus, HGF represents a potential therapeutic target for inhibiting the inflammatory response in adipose tissue and improving IR.

## HGF signaling in cancer

Activation of the HGF/c-Met signaling pathway is a hallmark of cancer cells and both HGF and c-Met have emerged as therapeutic targets. HGF, produced by some cancer cells, stimulates c-Met, through activation of the autocrine signaling system. Alternatively, HGF present in the tumor microenvironment activates c-Met receptors displayed on the surface of cancer cells, through the activation of the paracrine signaling system. The origin of HGF present in the tumor microenvironment is unknown and HGF could be synthesized by a variety of tissues throughout the body, such as the liver itself or by the adipose tissue. Recently, adipose tissue has been shown to be related to the increased prevalence and aggressiveness of some types of cancers (85–87). HGF is known to promote proliferation, migration, invasion and survival of cancer cells, and to confer resistance to therapy. Thus HGF is a possible link between obesity and cancer.

While researchers are currently investigating the relationship between HGF and cancer, some properties of HGF in the tumor microenvironment should be considered. Tumor stromal cells (macrophages, inflammatory cells, endothelial cells, and cancer-associated fibroblasts) secrete a variety of growth factors, chemokines and cytokines promoting a pro-inflammatory microenvironment for wound healing (88, 89). In fact, tumors could be considered wounds that do not heal (90). HGF is an important component of the fibroblast secretome (91), and has been shown to stimulate cancer cell invasiveness (88, 89), and promote the epithelial-mesenchymal transition (EMT), cell scattering and migration (52, 92–94).

As evidenced by the embryonic lethality in mice deficient in HGF or c-Met, it is thought that HGF/c-Met signaling stimulates the EMT, and plays a major role in embryogenesis, organogenesis, tissue repair and wound healing. As a growth factor, HGF promotes the growth and survival of cancer cells, increases tumor aggressiveness, stimulates metastasis and is associated with resistance to therapy (95, 96).

Recently, established colon cancer cell lines and primary colon tumors have been shown to produce large amounts of HGF (97). High levels of HGF were also observed in the serum and tumor tissue of stage II and III colon cancer patients, and especially in patients with lymph node and liver metastasis (98, 99). Moreover, increased HGF levels have been correlated with lymph node metastasis and relapse in individuals with breast cancer (100, 101), multiple myeloma (102), and myeloid leukemia (103).

Sensitivity to anticancer treatment is largely influenced by stromal cells, and HGF derived from these cells is a notable factor that provides resistance to target drugs (104). In general, cancer cells do not produce HGF, themselves. Rather, cancer-associated fibroblasts are primarily responsible for HGF production, and as a result activate c-Met paracrinally. Thus, HGF might be an important stromal cell component of the tumor microenvironment (105–108).

Unfortunately, conventional cancer treatment (radiotherapy or chemotherapy), is not able to distinguish a malignant cell from a normal one. Targeted therapies have the ability to circumvent this apparent lack of specificity. In fact, drugs that can block specific cancer cell dependent pathways such as: EGFR, BRAF, HER2, and HGF/c-Met signaling are being developed, and are predicted to have fewer side effects. Previous studies have shown that cells that undergo EMT are more resistant to cell death (109, 110), thus it is possible that the HGF-induced EMT is responsible for the increased resistance to therapy. In fact, in lung cancer cells with c-Met amplification, HGF has been shown to be responsible for the observed resistance to anti-MET treatment (111, 112). Thus, HGF production by the stromal cells inhibits the response to MET kinase inhibitors, as well as other signaling pathways (111). Additionally, EMT is also responsible for the acquisition of a stem cell phenotype, which is metabolically altered and markedly resistant to therapies. Moreover, HGF induces Wnt signaling in colon cancer cells, resulting in a cancer stem cell phenotype *in vitro* and *in vivo* (113). Lastly, activation of HGF/c-Met signaling also contributes to the cancer stem cell phenotype in other types of cancer such as: gliomas (114, 115), colon cancer (107), head and neck cancer (116), prostate cancer (117) and pancreatic cancer (118).

The data related to the HGF/c-Met signaling pathway and cancer suggests that the pathway inhibition may stunt the growth and progression of cancers. Thus, inhibition of HGF synthesis or activity in stromal cells may be an effective approach for future targeted cancer therapy.

## Conclusion and prospects

It has been nearly three decades since human HGF cDNA was successfully cloned (52). Since that time to the present day, the biological functions of the HGF/c-Met axis have been extensively investigated. The results presented from the concerted efforts of numerous laboratories have provided compelling evidence for the essential physiologically relevant functions of HGF, as well as its therapeutic potential.

The liver-originated hormone, HGF, considered a hepatokine, can potentially interfere with energy metabolism (37, 39). Similar to what was observed with adipokines and myokines, HGF has uncertain mixed functions, which can improve the metabolic profile of type 2 diabetes or induce IR (14, 119). Future investigations into the physiological role of liver-derived factors will likely yield potential biomarkers and/or lead to the discovery of novel therapies against type 2 diabetes and other metabolic complications.

Although the great amount of evidence has suggested that increased levels of HGF are associated with the manifestation of IR, this review has highlighted some of the beneficial effects stemming from HGF activity observed in obesity. Additionally, due to the fact that HGF markedly increases glucose metabolism and transport in myocytes and adipocytes, future research needs to focus on elucidating the details of how HGF contributes to the protective mechanism against the development of IR, in the liver, and possibly other tissues, such as skeletal muscle and WAT. Further support, for HGF being utilized as a therapeutic target in the treatment of IR, comes from its role in β-cell hyperplasia, and subsequent hyperinsulinemia.

Beyond emphasizing the proposed utility of HGF as potential therapeutic target in IR, this review also discussed how maternal β-cell adaptation, during pregnancy, depends on the HGF/c-Met signaling pathway, and that impairment of this pathway can lead to the onset of GDM. Regarding obesity-induced IR, which is strongly associated with an inflammatory state, HGF also emerges as a positive factor. This is because HGF has the ability to blunt inflammation in adipose tissue, by increasing M2 macrophage polarization, and consequently augmenting the expression of IL-10, both of which are positively associated with insulin sensitivity (see Figure 4). Additionally, the HGF/c-Met pathway plays a key role in the onset and progression of human cancers, and pathway activation is associated with a poor prognosis. While HGF may serve as the link between obesity and cancer, it should be noted that given the association of HGF levels with tumor progression, metastasis and less favorable response to cancer treatment, employing HGF for the modulation of IR or treating diabetes may be significantly limited by its cancer-promoting properties.

Future research in the area should focus on understanding the regulatory mechanisms, and developing inhibitors and activators for the modulation of endogenous HGF levels. In fact, innovative mechanisms for controlling HGF expression and/or activity could generate unique therapies for the treatment of IR and diabetes.

## Author contributions

All authors listed have made a substantial, direct and intellectual contribution to the work, and approved it for publication.

### Conflict of interest statement

The authors declare that the research was conducted in the absence of any commercial or financial relationships that could be construed as a potential conflict of interest.
